# Circulating omentin concentration increases after weight loss

**DOI:** 10.1186/1743-7075-7-27

**Published:** 2010-04-09

**Authors:** José María Moreno-Navarrete, Victoria Catalán, Francisco Ortega, Javier Gómez-Ambrosi, Wifredo Ricart, Gema Frühbeck, José Manuel Fernández-Real

**Affiliations:** 1 Service of Diabetes, Endocrinology and Nutrition, Institut d'Investigació Biomèdica de Girona (IdIBGi) Hospital Dr Josep Trueta, Girona, 17007, CIBEROBN (CB06/03/010), Spain; 2 Department of Endocrinology & Metabolic Research Laboratory, Clínica Universitaria de Navarra Pamplona, 31008, CIBEROBN (CB06/03/1014), Spain; 3 Instituto de Salud Carlos III (ISCIII) Madrid, 28029, Spain

## Abstract

**Background:**

Omentin-1 is a novel adipokine expressed in visceral adipose tissue and negatively associated with insulin resistance and obesity. We aimed to study the effects of weight loss-induced improved insulin sensitivity on circulating omentin concentrations.

**Methods:**

Circulating omentin-1 (ELISA) concentration in association with metabolic variables was measured in 35 obese subjects (18 men, 17 women) before and after hypocaloric weight loss.

**Results:**

Baseline circulating omentin-1 concentrations correlated negatively with BMI (r = -0.58, p < 0.001), body weight (r = -0.35, p = 0.045), fat mass (r = -0.67, p < 0.001), circulating leptin (r = -0.7, p < 0.001) and fasting insulin (r = -0.37, p = 0.03). Circulating omentin-1 concentration increased significantly after weight loss (from 44.9 ± 9.02 to 53.41 ± 8.8 ng/ml, p < 0.001). This increase in circulating omentin after weight loss was associated with improved insulin sensitivity (negatively associated with HOMA value and fasting insulin, r = -0.42, p = 0.02 and r = -0.45, p = 0.01, respectively) and decreased BMI (r = -0.54, p = 0.001).

**Conclusion:**

As previously described with adiponectin, circulating omentin-1 concentrations increase after weight loss-induced improvement of insulin sensitivity.

## Background

The communication between adipose tissue and other biological systems is accomplished through the expression of a large number of bioactive mediators that are collectively called 'adipokines' [1]. Adipokines play important roles in the pathogenesis of insulin resistance and associated metabolic complications such as dyslipidemia, hypertension, and premature heart disease [2].

Omentin-1 is a novel 34 kDa adipokine that is preferentially produced by visceral adipose tissue compared with subcutaneous adipose tissue [3,4]. Omentin-1 was also identified in human epicardial fat [5]. *In vitro *experiments revealed that treatment with recombinant omentin-1 enhanced insulin-stimulated glucose uptake in human subcutaneous and omental adipocytes, triggering Akt signaling in both the absence and presence of insulin [4]. Circulating omentin-1 correlated negatively with BMI, leptin, waist circumference, fasting insulin, and HOMA and positively with adiponectin and HDL in one study [6].

Weight loss is considered a key intervention to reduce the concentrations of proinflammatory cytokines and to increase circulating adiponectin levels [7,8]. No information is available concerning the effects of weight loss on circulating omentin-1 concentrations. Thus, we aimed to evaluate the effect of weight loss-induced improvement in insulin sensitivity on circulating omentin-1 concentrations.

## Methods

### Recruitment of participants

Thirthy-five Caucasian obese volunteers (17 females, 18 males) attending the Endocrinology Department at the University Clinic of Navarra were recruited. Patients underwent a clinical assessment including medical history, physical examination, body composition analysis, co-morbidity evaluation as well as nutritional interviews performed by a multidisciplinary consultation team. All subjects were non-smokers. Patients with signs of infection were excluded. Obese patients were not receiving statins or antidiabetic medication. The blood samples were taken in follicular stage of the menstrual cycle in pre-menopausal women.

Weight loss was achieved by prescription of a diet (in these subjects) providing a daily energy deficit of 500-1000 kcal/d as calculated from the determination of the resting energy expenditure through indirect calorimetry (Vmax29, SensorMedics Corporation, Yorba Linda, California) and multiplication by 1.4 as indicated for sedentary individual's to obtain the patient's total energy expenditure. This hypocaloric regime allows a safe and steady weight loss of 0.5-1.0 kg/wk when followed and supplied 30, 54 and 16% of energy requirements in the form of fat, carbohydrates and protein, respectively. The length of the hypocaloric diet was four months.

Body fat was estimated by air-displacement-plethysmography (Bod-Pod^®^, Life Measurements, Concord, California, USA).

The institutional review board of the institution approved the protocol, so we certify that all applicable institutional regulations concerning the ethical use of information and samples from human volunteers were followed during this research.

#### Analytical methods

Plasma glucose was analyzed by an automated analyzer (Roche/Hitachi Modular P800) as previously described [9]. Insulin was measured by means of an enzyme-amplified chemilumi-nescence assay (IMMULITE^®^, Diagnostic Products Corp., Los Angeles, CA, USA). To estimate insulin resistance, the HOMA index was calculated as fasting insulin concentration (μU/mL) × fasting glucose concentration (mmol/L)/22.5.

Total serum cholesterol was measured through the reaction of cholesterol esterase/cholesterol oxidase/peroxidase, using a Hitachi 747. HDL cholesterol was quantified after precipitation with polyethylene glycol at room temperature [10]. LDL cholesterol was calculated using the Friedewald formula. Total serum triglycerides were measured through the reaction of glycerol/phosphate/oxidase and peroxidase [11].

Serum omentin-1 concentrations were measured using manual omentin-1 (human) detection set (ELISA) (APOTECH^® ^Corporation, Switzerland). Serum samples were diluted and assayed according to the manufacturer's instructions. Intra- and interassay coefficients of variation were between 5% and 10%. The detection limit of the assay is 0.4 ng/ml (range 0.78 to 50 ng/ml). The antibodies used in this detection Set are specific for measurement of natural and recombinant human omentin-1.

### Statistical methods

Descriptive results of continuous variables are expressed as mean ± SD. Relation between variables was tested using Spearman's test. Paired t tests were used to for comparisons of quantitative variables across weight loss. For a given value of p = 0.05, the study had a 99% power to detect significant correlations between parameters in the whole sample of subjects in a bilateral test (n = 35). The analyses were performed using the program SPSS (version 11.0).

## Results

Table 1 shows the main characteristics of the subjects included in this study and the correlations between circulating baseline omentin-1 concentrations and baseline study variables.

**Table 1:** Subjects' characteristics and linear correlation analyses between circulating baseline omentin-1 concentrations and baseline parameters in all subjects.

	All subjects (n = 35)				
	Baseline	After weight loss	p	r*	p*
**Age (years)**	42.4 ± 15.2			0.23	0.2
**Body Mass Index (kg/m^2^)**	33.3 ± 4.2	28.9 ± 4.4	**<0.001**	-0.58	**<0.001**
**Body weight (kg)**	95.4 ± 17.5	82.4 ± 14.7	**<0.001**	-0.35	**0.045**
**Fat mass (%)**	41.8 ± 8.2	33.9 ± 9.9	**<0.001**	-0.67	**<0.001**
**Waist (cm)**	106.4 ± 11.4	96.4 ± 11.7	**<0.001**	-0.15	0.4
**Hip (cm)**	112.8 ± 10.3	104.4 ± 9.4	**<0.001**	-0.58	**<0.001**
**Waist to hip ratio**	0.95 ± 0.08	0.92 ± 0.07	**<0.001**	0.28	0.1
**Fasting glucose (mmol/l)**	5.2 ± 0.5	5 ± 0.43	**0.04**	0.23	0.2
**Fasting triglycerides (mmol/l)**	1.23 ± 0.5	0.92 ± 0.36	**0.001**	0.17	0.3
**HDL-cholesterol (mmol/l)**	1.37 ± 0.3	1.28 ± 0.28	0.05	-0.08	0.7
**LDL-cholesterol (mmol/l)**	3.3 ± 0.8	2.7 ± 0.59	**<0.001**	0.28	0.1
**Total cholesterol (mmol/l)**	5.2 ± 0.9	4.45 ± 0.61	**<0.001**	0.3	0.08
**Fasting insulin (mU/l)**	16.1 ± 8.6	12.06 ± 6.4	**0.005**	-0.37	**0.03**
**HOMA value**	3.6 ± 2.04	2.6 ± 1.4	**0.009**	-0.3	0.08
**Circulating leptin (ng/ml)**	35.7 ± 22.2	13.2 ± 18.2	**<0.001**	-0.7	**<0.001**
**Circulating omentin-1 (ng/ml)**	44.9 ± 9.02	53.4 ± 8.8	**<0.001**		

*Correlation between circulating baseline omentin concentrations and baseline parameters.

Baseline and post-weight loss omentin-1 concentrations were significantly higher in men than women (48.1 ± 8.3 *vs*. 40.3 ± 8.6 ng/ml in baseline serum, p = 0.009 and 56.4 ± 8.7 *vs*. 49.5 ± 8.03 ng/ml in post-weight loss serum, p = 0.01). Baseline circulating omentin-1 concentrations correlated negatively with BMI (r = -0.67, p = 0.002), body weight (r = -0.57, p = 0.01), fat mass (r = -0.48, p = 0.04), waist circumference (r = -0.47, p = 0.045), hip circumference (r = -0.52, p = 0.03) and circulating leptin (r = -0.65, p = 0.003) among men (Table 2); and with BMI (r = -0.69, p = 0.002), body weight (r = -0.64, p = 0.006), fat mass (r = -0.63, p = 0.007), hip circumference (r = -0.67, p = 0.003), circulating leptin (r = -0.74, p = 0.001) and fasting insulin (r = -0.64, p = 0.005) and HOMA (r = -0.55, p = 0.02) among women (Table 3). In a multiple linear regression analyses, BMI (p = 0.01) and sex (p = 0.01) were two independent contributors to circulating omentin variance after adjusting by fasting insulin. When we studied the participants according to sex, we found in both cases that BMI (p = 0.038 in men and p = 0.01 in women) was an independent contributor to circulating omentin variance after adjusting by fasting insulin (Table 4).

**Table 2:** Subjects' characteristics and linear correlation analyses between circulating baseline omentin-1 concentrations and baseline parameters in men.

	Men (n = 18)				
	Baseline	After weight loss	P	r*	p*
**Age (years)**	41.7 ± 15.1			0.28	0.25
**Body Mass Index (kg/m^2^)**	33.3 ± 4.23	28.9 ± 4.4	**<0.001**	-0.67	**0.002**
**Body Weight (kg)**	102.6 ± 14.7	88.8 ± 13.2	**<0.001**	-0.59	**0.01**
**Fat mass (%)**	36.2 ± 5.9	27.3 ± 7.6	**<0.001**	-0.48	**0.04**
**Waist (cm)**	111.2 ± 7.2	99.1 ± 8.6	**<0.001**	-0.47	**0.045**
**Hip (cm)**	111.4 ± 8.37	104 ± 8.9	**<0.001**	-0.52	**0.03**
**Waist to hip ratio**	1 ± 0.05	0.95 ± 0.05	**<0.001**	0.11	0.6
**Fasting glucose (mmol/l)**	5.3 ± 0.27	5.1 ± 0.45	0.09	0.35	0.17
**Fasting triglycerides (mmol/l)**	1.37 ± 0.59	0.92 ± 0.37	**0.002**	-0.05	0.8
**HDL-cholesterol (mmol/l)**	1.26 ± 0.25	1.2 ± 0.27	0.17	-0.14	0.6
**LDL-cholesterol (mmol/l)**	3.5 ± 0.8	2.8 ± 0.6	**0.001**	-0.05	0.8
**Total cholesterol (mmol/l)**	5.4 ± 0.9	4.4 ± 0.65	**<0.001**	-0.07	0.8
**Fasting insulin (mU/l)**	14.9 ± 6.9	9.7 ± 5.6	**0.005**	-0.38	0.13
**HOMA value**	3.5 ± 1.6	2.2 ± 1.2	**0.005**	-0.37	0.14
**Circulating Leptin (ng/ml)**	23.1 ± 11.7	13.4 ± 7.8	**<0.001**	-0.65	**0.003**
**Circulating omentin-1 (ng/ml)**	48.6 ± 8.3	56.4 ± 8.9	**0.003**		

*Correlation between circulating baseline omentin concentrations and baseline parameters.

**Table 3:** Subjects' characteristics and linear correlation analyses between circulating baseline omentin-1 concentrations and baseline parameters in women.

	Women (n = 17)				
	Baseline	After weight loss	P	r*	p*
**Age (years)**	43.3 ± 15.6			0.2	0.4
**Body Mass Index (kg/m^2^)**	33.8 ± 6.45	29.2 ± 5.1	**<0.001**	-0.69	**0.002**
**Body Weight (kg)**	87.7 ± 17.4	75.6 ± 13.4	**<0.001**	-0.64	**0.006**
**Fat mass (%)**	47.6 ± 5.7	40.8 ± 6.8	**<0.001**	-0.63	**0.007**
**Waist (cm)**	101.5 ± 12.9	93.6 ± 14.06	**<0.001**	-0.44	0.08
**Hip (cm)**	114.2 ± 12.2	104.8 ± 10.2	**<0.001**	-0.67	**0.003**
**Waist to hip ratio**	0.88 ± 0.07	0.89 ± 0.08	**<0.001**	0.13	0.6
**Fasting glucose (mmol/l)**	5.04 ± 0.6	5.01 ± 0.38	**0.047**	-0.07	0.8
**Fasting triglycerides (mmol/l)**	1.06 ± 0.45	0.92 ± 0.36	**0.001**	0.1	0.7
**HDL-cholesterol (mmol/l)**	1.5 ± 0.35	1.37 ± 0.27	0.47	0.21	0.4
**LDL-cholesterol (mmol/l)**	3.1 ± 0.86	2.7 ± 0.6	**0.001**	0.35	0.2
**Total cholesterol (mmol/l)**	5.06 ± 0.97	4.5 ± 0.57	**<0.001**	0.43	0.09
**Fasting insulin (mU/l)**	12.03 ± 9.6	9.6 ± 7.4	0.05	-0.64	**0.005**
**HOMA value**	2.54 ± 2.2	2 ± 1.6	0.05	-0.55	**0.02**
**Circulating Leptin (ng/ml)**	32.5 ± 7.8	12.8 ± 3.8	**<0.001**	-0.74	**0.001**
**Circulating omentin-1 (ng/ml)**	41.1 ± 8.3	50.2 ± 7.6	**0.001**		

*Correlation between circulating baseline omentin concentrations and baseline parameters.

**Table 4:** Multiple linear regression analyses with baseline circulating omentin-1 as dependent variable before weight loss

	All subjects		Men		Women	
	Beta	P	Beta	p	Beta	p
**Sex**	-2.76	**0.01**	---		---	
**Body Mass Index (kg/m^2^)**	-2.74	**0.01**	-2.3	**0.038**	-2.9	**0.01**
**Fasting insulin (mU/l)**	-0.27	0.8	0.09	0.9	-0.71	0.5
***Adjusted R Square***	**0.39**		**0.28**		**0.36**	

**Beta **is the standardized regression coefficient, which allows evaluating the relative significance of the each independent variable in multiple linear regression analyses.

**Adjusted R square **express the percentage of the variance explained by the independent variables in the different models (i.e., 0.50 is 50%).

Baseline and post-weight loss leptin concentrations were significantly higher in women than men (48.27 ± 21.1 *vs*. 26.04 ± 17.25 ng/ml in baseline serum, p = 0.001 and 29.57 ± 18.5 *vs*. 9.8 ± 10.5 ng/ml in post-weight loss serum, p = 0.01). Baseline circulating leptin concentrations correlated with obesity measures, BMI (r = 0.69, p = 0.001 in men and r = 0.67, p = 0.003 in women), waist circumference (r = 0.65, p = 0.001 in men and r = 0.55, p = 0.01 in women), hip circumference (r = 0.71, p < 0.001 in men and r = 0.69, p = 0.001 in women) and fat mass (r = 0.62, p = 0.006 in men, and r = 0.67, p = 0.003 in women), fasting insulin (r = 0.53, p = 0.02 in men, and r = 0.77, p < 0.001 in women) and HOMA (r = 0.57, p = 0.01 in men, and r = 0.72, p = 0.001 in women) and inversely with circulating omentin (r = -0.65, p = 0.003 in men, and r = -0.74, p = 0.001 in women).

**Figure 1:**
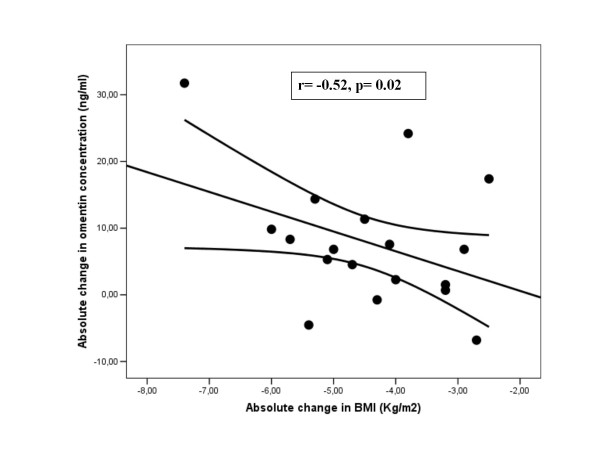
**Correlation between absolute change in omentin-1 and absolute change in BMI (kg/m^2^) in men (r = -0.52, p = 0.02, n = 18)**.

**Figure 2:**
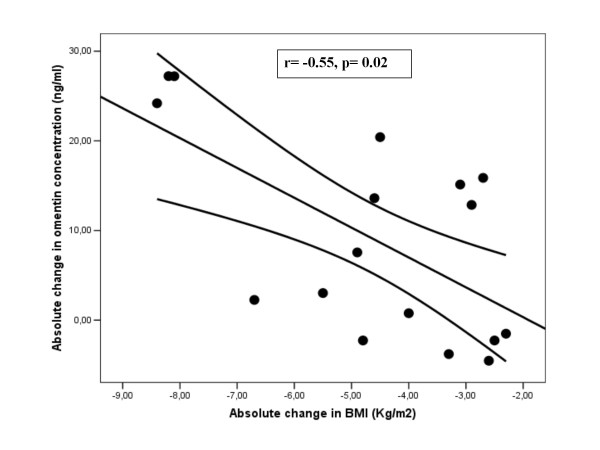
**Correlation between absolute change in omentin-1 and absolute change in BMI (kg/m^2^) in women (r = -0.55, p = 0.02, n = 17)**.

After weight loss, circulating omentin-1 concentration increased significantly in the study subjects (Table 1). The increase in circulating omentin concentrations was associated with the decrease of fasting insulin (r = -0.45, p = 0.01), HOMA index (r = -0.42, p = 0.02), BMI (r = -0.47, p = 0.003) and circulating leptin (r = -0.4, p = 0.025) (Table 5 and Figure 1 and 2). Circulating leptin concentration decreased significantly after weight loss in both men and women (Table 1). The decrease in circulating leptin was significantly associated with the decrease of BMI (r = 0.67, p = 0.001), fasting insulin (r = 0.52, p = 0.044), HOMA (r = 0.54, p = 0.04) and circulating omentin (r = - 0.49, p = 0.04) in men and only with the decrease of BMI (r = 0.5, p = 0.02) in women.

**Table 5:** Linear correlation analyses between the changes of log transformed omentin-1 concentration and the change of study variables after weight loss.

	All subjects (n = 35)		Men (n = 18)		Women (n = 17)	
	r	P	R	p	r	p
**Body Mass Index (kg/m^2^)**	-0.47	**0.003**	-0.52	**0.02**	-0.55	**0.02**
**Body Weight (kg)**	-0.44	**0.01**	-0.47	**0.04**	-0.53	**0.03**
**Fat mass (%)**	-0.29	0.09	-0.27	0.2	-0.31	0.2
**Waist (cm)**	-0.04	0.8	-0.28	0.2	0.2	0.4
**Hip (cm)**	-0.31	0.06	-0.3	0.2	-0.38	0.1
**Waist to hip ratio**	0.19	0.2	-0.2	0.4	0.4	0.1
**Fasting glucose (mmol/l)**	-0.04	0.8	-0.28	0.2	0.12	0.6
**Fasting triglycerides (mmol/l)**	-0.1	0.6	-0.3	0.2	0.2	0.4
**HDL-cholesterol (mmol/l)**	0.37	**0.03**	0.21	0.4	0.43	0.08
**LDL-cholesterol (mmol/l)**	0.04	0.8	-0.24	0.3	0.37	0.1
**Total Cholesterol (mmol/l)**	0.09	0.6	-0.35	0.1	0.5	0.06
**Fasting insulin (mU/l)**	-0.45	**0.01**	-0.5	**0.03**	-0.53	**0.03**
**HOMA index**	-0.42	**0.02**	-0.48	**0.04**	-0.46	0.08
**Circulating Leptin (ng/ml)**	-0.4	**0.025**	-0.49	**0.04**	-0.26	0.34

## Discussion

The main finding of this study was the observed increase in circulating omentin-1 concentration after weight loss and its association with the improvement in insulin sensitivity. This finding is in agreement with the reported effects of hyperinsulinemic inhibition of omentin-1 production in healthy subjects [12]. Thus, insulin-downregulated omentin-1 production could be behind the inverse relationship between circulating omentin-1 and obesity.

Circulating omentin-1 was weakly correlated with waist in men and no association was found in women, in contrast to the correlation with hip circumference. We may speculate that hyperinsulinemic conditions and peripheral adiposity may reduce omentin-1 production in morbid obese subjects.

Circulating omentin-1 concentrations measured by ELISA were higher in men than women, although the increase of omentin-1 after weight loss was similar in both groups. Using quantitative western blotting, Batista *et al*. [6] found increased omentin-1 concentration in women (N = 21) compared with men (N = 18) in genetically homogeneous population of Old order Amish. Even though these authors concluded that more studies were required to address this apparently divergent regulation of omentin-1 between men and women. Many adipokines have been found to exhibit a sexual dimorphism. Both leptin and adiponectin are increased in serum of women compared with men. We corroborated that in women leptin concentration is higher than in men and the relationship between circulating leptin and obesity, decreasing significantly serum leptin concentration after weight loss [13]. The strong relationship between baseline circulating leptin and omentin-1 in men and women was other evidence about the closed association between omentin-1 and adiposity measures. This observation has been explained on the basis of different fat amounts and the influences of sex hormones. Plasma RBP4 concentrations, however, exhibit an opposite pattern [14].

We compared metabolic baseline parameters between men and women, and we found that in women fat mass and circulating leptin were significantly higher than in men (47.6 ± 5.7 vs. 36.2 ± 5.9%, p < 0.0001 and 48.2 ± 21.1 vs. 26.1 ± 17.2 ng/ml, p = 0.001, respectively). The increased association among fat mass, circulating leptin and baseline circulating omentin-1 may explain the sex differences in omentin-1 concentration in this study. Supporting these findings, Tan *et al *reported that omental adipose tissue omentin-1 mRNA expression and protein concentrations were negatively associated with circulating 17β-estradiol [12].

The associations of omentin-1 with metabolic variables (6, 12, current report) were similar to those described for adiponectin [4], sharing common insulin sensitizing activities.

The weak correlation of the change of omentin-1 vs. the change of HOMA (IR) and BMI in each could be caused by the small number of subjects of this study. The lack of correlation of the change of omentin-1 vs. the change in other variables could be explained because the decrease of BMI was the main independent contributors to circulating omentin-1 variation.

In conclusion, these preliminary findings showed that the increase of omentin-1 concentration run in parallel with the increase of insulin sensitivity after weight loss.

## Competing interests

The authors declare that they have no competing interests.

## Authors' contributions

JMM-N carried out the biochemical analyses and immunoassays, participates in acquisition, analysis and interpretation of data and drafted the manuscript. VC, FO, JG-A: Participate in acquisition of data and in the biochemical and clinical determinations, and reviewed the manuscript. WR, GF: Participate in design and reviewed the manuscript critically for important intellectual content. JMF-R: Participate in design and coordination, and helped to draft the manuscript and reviewed the manuscript critically for important intellectual content. All authors read and approved the final manuscript.
